# Reductive faceted photocatalytic nanocoating for uranium extraction from seawater†

**DOI:** 10.1039/d5ra02388b

**Published:** 2025-07-23

**Authors:** Chen Xie, Yizhi Zeng, Bohao Zhao, Ning Lv, Guiming Chen

**Affiliations:** a ^a^, High-Tech Institute of Xi'an Xi'an Shaanxi 710025 China 1010280093@qq.com

## Abstract

Photocatalytic technology, as an emerging method for uranium extraction from seawater, has garnered significant attention due to its potential for high efficiency, low cost, and environmental sustainability. However, most existing photocatalytic materials are in powder form, which not only limits their effective recovery in natural seawater environments but also indicates that their reductive performance still has considerable room for improvement. To address these challenges, this study proposes a strategy to construct photocatalytic coatings on organic plastic films, enabling material recyclability. Additionally, through crystal facet engineering, the specific facets of the photocatalyst were exposed, forming electron-rich surfaces that enhance the tendency of atomic nuclei to lose electrons. This modification significantly strengthened the generation of reductive species, thereby improving the efficiency of photocatalytic reduction to tetravalent uranium species at the interface. Consequently, the uranium extraction performance from seawater was enhanced. Compared to existing P25-based recyclable materials, this method achieved approximately 1.64 times higher uranium extraction efficiency and maintained over 85% extraction efficiency after seven cycles of reuse. This study provides a simple and efficient new approach for uranium extraction from seawater, demonstrating considerable potential for practical applications.

## Abstract 2

Photocatalytic technology, as an emerging method for uranium extraction from seawater, has garnered significant attention due to its potential for high efficiency, low cost, and environmental sustainability.

## Graphical abstract

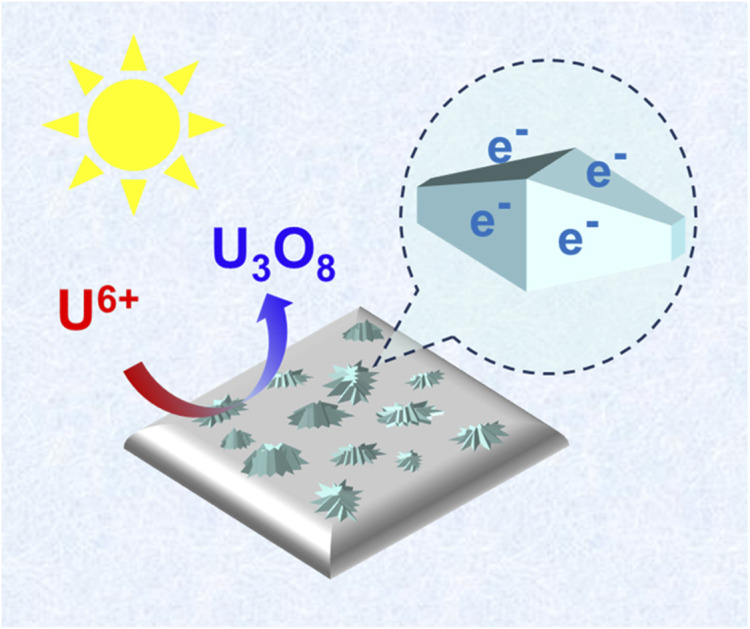

## Introduction

Uranium extraction from seawater is regarded as one of the seven separation technologies that could change the world,^1^ playing a crucial role in ensuring sustainable energy supply and environmental development. However, the mainstream uranium extraction technologies currently rely on organic ligand adsorption,^2–5^ which is highly susceptible to interference from microorganisms^6^ and other pollutants in complex marine environments,^7^ significantly reducing their practical effectiveness. In contrast, photocatalytic uranium extraction from seawater generates reactive species under light excitation, which not only effectively inhibits the growth and reproduction of microorganisms but also accelerates the uranium extraction process. As a result, it is considered a more ideal and sustainable green solution.

In recent years, researchers have developed various promising photocatalytic materials for uranium extraction from seawater, including graphitic carbon nitride,^8,9^ copper oxide^10,11^ and metal–organic frameworks.^12–14^ However, uranium extraction from seawater is a large-scale engineering challenge that requires careful cost considerations. The high costs associated with these materials limit their potential for large-scale production. In comparison, titanium dioxide (TiO_2_) stands out as the most representative photocatalyst due to its low cost, stability, and ease of synthesis, and it has already been scaled up for industrial production and application. Moreover, studies have shown that industrial-grade P25 titanium dioxide exhibits significantly better uranium extraction performance than most other materials,^15,16^ making it a relatively ideal photocatalyst for seawater uranium extraction. Nevertheless, photocatalytic materials represented by P25 titanium dioxide still face two major challenges: first, the carrier separation efficiency of homogeneous nanoparticles is relatively low,^17,18^ as excessive recombination of electrons and holes results in fewer electrons being transferred to the catalyst surface for uranium reduction; second, the powdered form of the catalyst makes it difficult to recover conveniently after uranium extraction,^19,20^ limiting its practical application in industrial processes.

To address these challenges, this study employed a hydrothermal method with surface inhibitors to synthesize titanium dioxide nanoparticles with exposed {101} facets, inducing electron enrichment on the outer crystal surfaces to improve carrier separation efficiency. By enhancing the interfacial reductive properties of the material, the photocatalytic uranium extraction capability from seawater was significantly improved. Additionally, the titanium dioxide nanoparticles were immobilized on the surface of thin films using a binder, resulting in a recyclable photocatalytic coating. Subsequently, this study systematically analyzed the photocatalytic uranium extraction performance, antimicrobial properties, and recyclability of the coating, and further explored its potential applications in other fields such as nuclear wastewater and groundwater treatment. This research provides a simple and efficient recyclable solution for photocatalytic uranium separation technology, offering valuable insights for future studies.

## Methods

### Materials and reagents

In this study, natural seawater was collected from the coastal waters near Hainan Province, China. To facilitate testing and minimize interference from other ions, a small amount of uranyl nitrate was added to the seawater to achieve a uranium concentration of 300 μg L^−1^, which is approximately 100 times higher than the natural uranium concentration in seawater. The P25, anatase, and rutile samples used in this experiment were purchased from XFNANO Materials Technology Co., Ltd, while other common reagents, such as potassium hydroxide, were obtained from Sinopharm Chemical Reagent Co., Ltd. The P25 used in this study is an industrial product with a phase composition of 60% anatase and 40% rutile.

### Synthesis of {101} TiO_2_ powder

To synthesize {101} TiO_2_ powder, 1 g of P25 powder was added to 50 mL of saturated NaOH solution and mixed thoroughly. The mixture underwent hydrothermal treatment at 160 °C for 28 hours. The resulting solid product was separated by centrifugation, washed, and dried. Subsequently, 500 mg of the solid product was dispersed in 50 mL of pure water and subjected to hydrothermal treatment at 180 °C for 20 hours. The final product was obtained by centrifugation, washing, and drying, resulting in {101} TiO_2_ powder.

### Preparation of recyclable catalyst coatings

Aluminum foil sheets measuring 3.5 cm × 3 cm were cut and evenly coated with a waterproof silicone adhesive. Then, 60 mg of catalyst powder was gently sprinkled onto the surface of the foil and spread evenly. After drying, the recyclable catalyst coating was obtained.

### Photocatalytic uranium extraction experiment

The recyclable catalyst coatings were immersed in 50 mL of uranium-spiked seawater with a uranium concentration of 300 μg L^−1^. The intensity of the xenon lamp was adjusted to 100 mW cm^−2^. Samples were collected before and after 1 hour of light irradiation, and the uranium extraction efficiency of the material was calculated based on the ratio of uranium concentrations before and after irradiation. After the experiment, the uranium-containing wastewater was collected, the uranium in the seawater continued to be adsorbed to less than 30 μg L^−1^ using an excess of {101} TiO_2_ powder, the adsorbed uranium solids were separated, and the wastewater was discharged according to the standard, while the solids were centrally disposed of by specialised hazardous chemical recycling companies.

### Characterization methods

The crystal morphology was characterised using field-emission transmission electron microscopy (TEM, Tecnai G2F30 S-TWIN, FEI, USA) at an accelerating voltage of 300 kV. Morphological images of the samples were obtained using scanning electron microscopy (SEM, SU8020, Hitachi, Japan) at an accelerating voltage of 5 kV. Elemental distribution images were acquired using energy-dispersive X-ray spectroscopy (EDX, EMAX mics2, HORIBA, Japan) at an accelerating voltage of 15 kV. The crystalline phase properties of the material were determined by X-ray diffraction (XRD, D8 Advance, Bruker, Germany). The generation of free radicals and holes was detected *via* electron paramagnetic resonance (EPR, A300-10/12, Bruker, Germany). The valence band spectra and elemental composition of the samples were characterised by X-ray photoelectron spectroscopy (XPS, Thermo ESCALAB 250Xi, Thermo Fisher, USA).

## Results and discussion

**Fig. 1:**
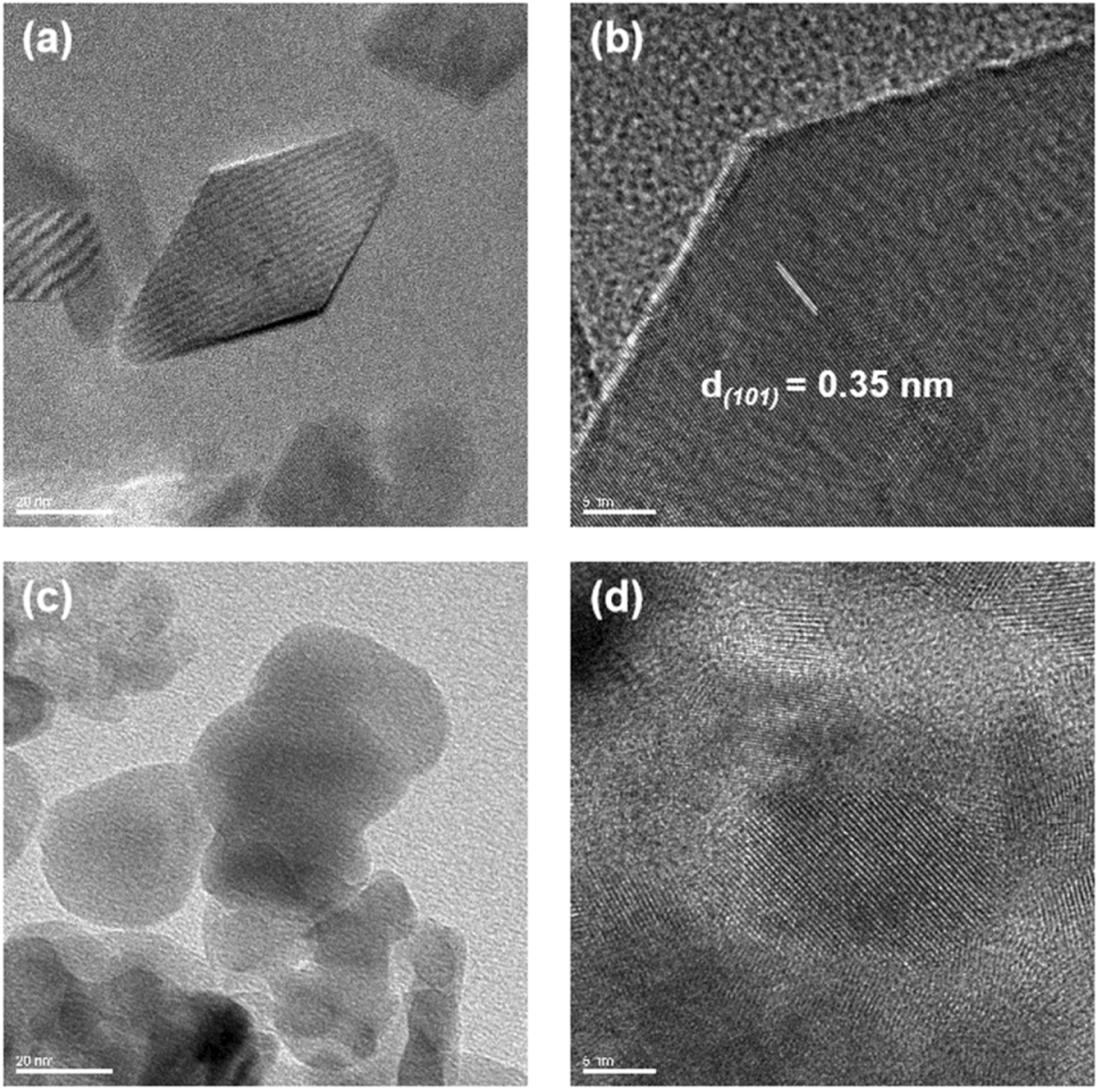
(a) TEM image and (b) HRTEM image of {101} TiO_2_ nanoparticles. (c) TEM image and (d) HRTEM image of P25 nanoparticles.

**Fig. 2:**
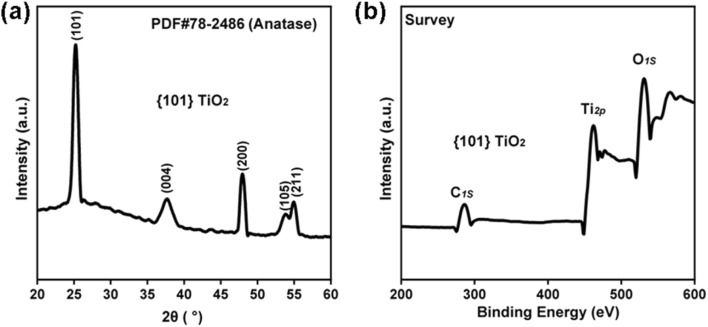
(a) XRD pattern and (b) XPS spectrum of {101} TiO_2_.

In this study, {101} TiO_2_ nanoparticles with exposed specific facets were successfully synthesized using a hydrothermal method with surface inhibitors. The results of the Brunauer–Emmett–Teller (BET) test showed that {101} TiO_2_ and P25 had similar specific surface areas (Fig. S1†), suggesting that the difference in catalytic performance between the two was mainly due to the interfacial factor rather than the specific surface area factor. Transmission electron microscopy (TEM) analysis revealed that the nanoparticles exhibit a double inverted pyramid shape (Fig. 1a) with well-defined faceted structures. The lattice fringe spacing was measured to be 0.35 nm (Fig. 1b), corresponding to the (101) orientation of anatase-phase titanium dioxide. This result is consistent with the XRD analysis shown in Fig. 2a, further confirming that the {101} TiO_2_ nanoparticles align with the crystal characteristics of the #78-2486 standard card. Additionally, the lattice fringes of {101} TiO_2_ displayed high continuity, indicating a high degree of crystallinity and the presence of exposed specific facets. In contrast, P25 nanoparticles were observed to have a spherical morphology (Fig. 1c) with randomly oriented lattice fringes (Fig. 1d). Although P25 nanoparticles also exhibited a high degree of crystallinity, they lacked the continuous faceted structure observed in {101} TiO_2_. XPS survey spectra further demonstrated that {101} TiO_2_ primarily consists of pure titanium dioxide (Fig. 2b), with no significant incorporation of surface inhibitors into its crystal structure. These findings indicate that {101} TiO_2_ nanoparticles with exposed specific facets were successfully synthesized in this study. Compared to conventional P25 nanoparticles, the {101} TiO_2_ nanoparticles exhibit superior crystal structure characteristics, providing a robust foundation for efficient photocatalytic reactions.

**Fig. 3:**
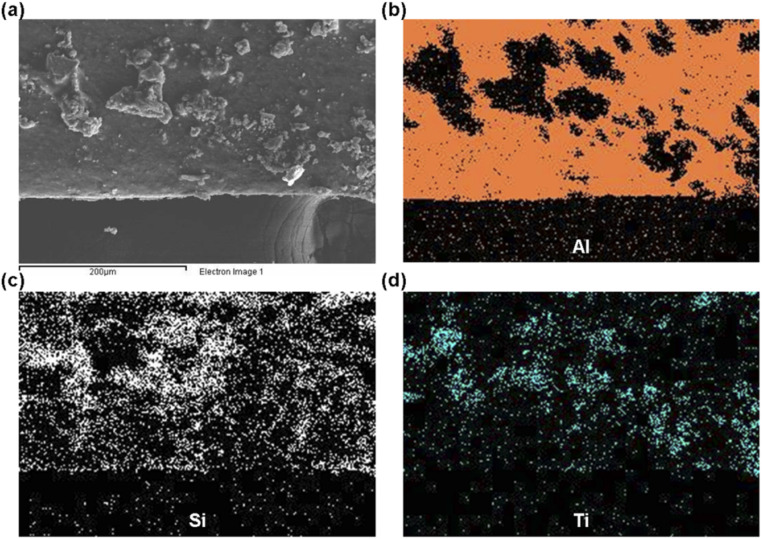
(a) SEM image of {101} TiO_2_ smear, along with its elemental distribution maps: (b) Al, (c) Si, (d) Ti.

To enhance the recyclability of photocatalytic materials for uranium extraction from seawater, this study applied a coating method to load {101} TiO_2_ nanoparticles onto the surface of aluminum foil. Scanning electron microscopy (SEM) results indicate that the {101} TiO_2_ nanoparticles adhere tightly to the substrate surface (Fig. 3a). Further elemental distribution analysis revealed that aluminum is mainly concentrated in relatively smooth regions (Fig. 3b), corresponding to the aluminum foil substrate. Silicon, however, is evenly distributed across the entire surface (Fig. 3c), indicating that the silica binder forms a uniform coating. The distribution of titanium is complementary to that of aluminum (Fig. 3d), further confirming that the particles observed in Fig. 3a are titanium dioxide particles. These findings demonstrate that this study successfully employed a simple method to achieve heavy loading of photocatalysts on the substrate surface, facilitating the convenient recovery of photocatalysts after uranium extraction from seawater.

**Fig. 4:**
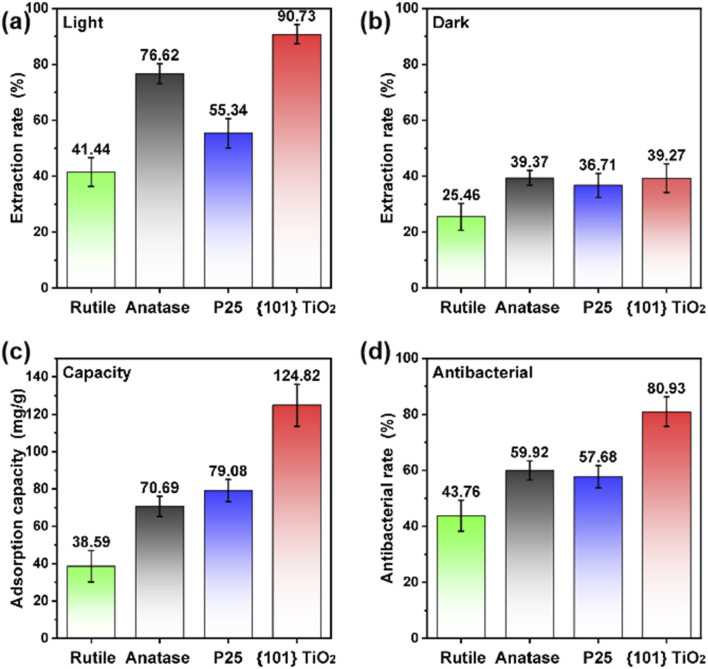
(a) Photocatalytic uranium extraction from seawater using the catalyst coating, (b) uranium adsorption under dark conditions, (c) uranium extraction capacity, and (d) antimicrobial performance.

This study systematically evaluated the seawater uranium extraction performance of photocatalytic coatings. Under light irradiation, the uranium extraction efficiencies of rutile, anatase, P25, and {101} TiO_2_ coatings were 41.44%, 76.62%, 55.34%, and 90.73%, respectively (Fig. 4a). These results indicate that titanium dioxide-based photocatalytic coatings are effective in extracting uranium from seawater under light conditions. Furthermore, compared to the rutile phase, the anatase phase exhibited higher efficiency in uranium extraction, and the exposure of {101} facets on the anatase phase further enhanced the extraction performance. Under dark conditions, the uranium adsorption efficiencies of rutile, anatase, P25, and {101} TiO_2_ coatings were 25.46%, 39.97%, 36.71%, and 39.27%, respectively (Fig. 4b). In addition, the seawater uranium extraction effect of the aluminium foil substrate and binder was analysed, and the results of Fig. S2† show that the substrate is basically not adsorptive to uranium, which proves that the seawater uranium extraction effect of the photocatalytic coating is related to the nature of its catalysts, and is not related to the substrate. These findings suggest that the uranium adsorption capacities of different nanoparticles are relatively similar and low, indicating that photocatalytic activity plays a dominant role in the uranium extraction process, while the intrinsic properties of nanoparticles have a limited impact on adsorption performance. Additionally, the uranium extraction capacities of different photocatalytic coatings were tested using uranium-spiked seawater with a concentration of 100 ppm. The results showed that {101} TiO_2_ exhibited the highest extraction capacity of 124.82 mg g^−1^ (Fig. 4c). Moreover, the antimicrobial performance of the photocatalytic coatings was evaluated, and {101} TiO_2_ demonstrated the highest antibacterial rate of 80.93% among all tested samples (Fig. 4d). In summary, the {101} TiO_2_ nanoparticle coating, with its exposed facets, exhibited superior performance in terms of uranium extraction rate, extraction capacity, and antimicrobial properties, highlighting its potential advantages for seawater uranium extraction applications.

**Fig. 5:**
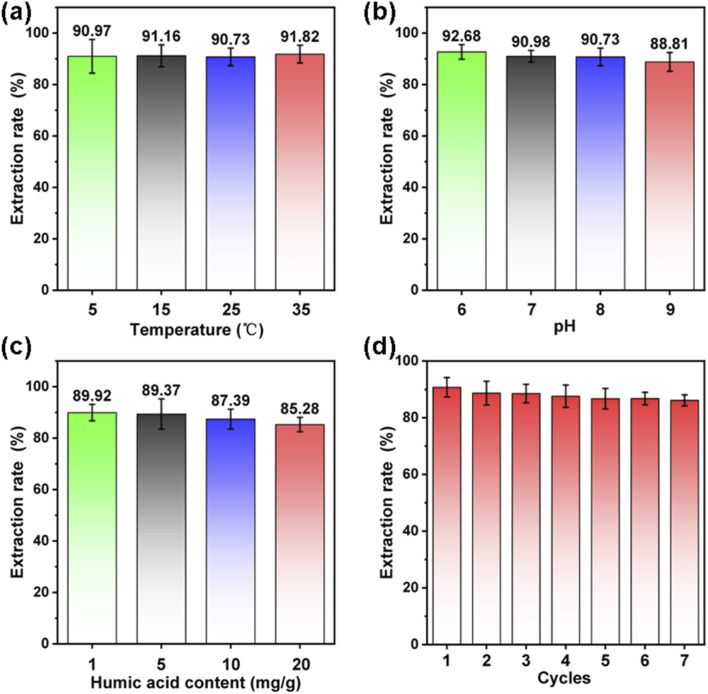
Effects of various factors on the photocatalytic uranium extraction from seawater using the catalyst coating: (a) temperature, (b) pH, (c) organic matter concentration, and (d) recycling.

The initial results indicate that the {101} TiO_2_ coating demonstrates relatively ideal performance in photocatalytic uranium extraction from seawater. To further evaluate its potential for practical applications, this study analyzed the effects of different environmental factors on its performance. By adjusting the solution temperature and pH to simulate variations in seawater environments, the results showed that the photocatalytic uranium extraction efficiency of the {101} TiO_2_ coating remained stable at approximately 90% within the temperature range of 5 °C to 35 °C (Fig. 5a) and a pH range of 6 to 9 (Fig. 5b), indicating that fluctuations in temperature and pH had minimal impact on its uranium extraction performance. Additionally, humic acid was added to seawater to simulate interference from varying concentrations of organic pollutants.^21,22^ The results revealed that in the concentration range of 1 to 20 mg g^−1^, the photocatalytic uranium extraction efficiency of the {101} TiO_2_ coating remained between 85.28% and 89.92% (Fig. 5c), suggesting that the concentration of organic matter had a limited effect on its catalytic performance. The study also tested the recyclability of the {101} TiO_2_ coating, showing that its photocatalytic uranium extraction efficiency consistently stayed above 86.1% over seven cycles of use (Fig. 5d). These findings demonstrate that the {101} TiO_2_ coating exhibits high stability in uranium extraction performance under fluctuating seawater conditions and maintains excellent efficiency across multiple reuse cycles.

**Fig. 6:**
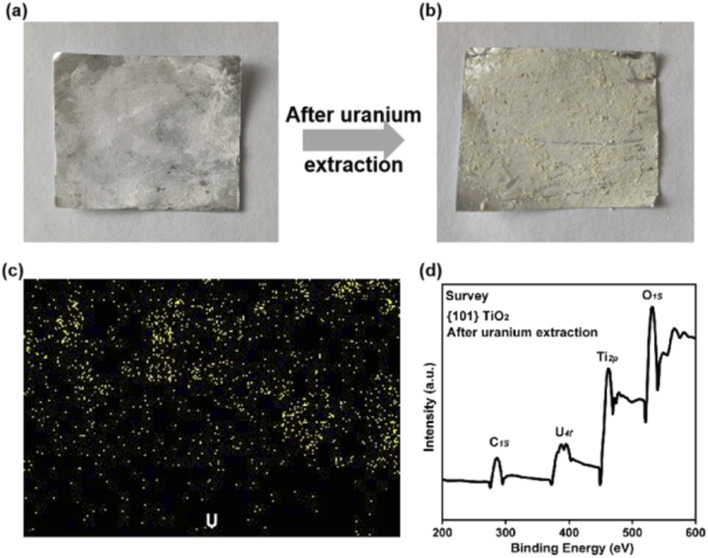
Photographs of the {101} TiO_2_ film (a) before and (b) after photocatalytic uranium extraction from seawater, (c) uranium element distribution map, and (d) XPS spectrum.

This study conducted an in-depth analysis of {101} TiO_2_ films after repeated use. The results showed that the surface powder of the original {101} TiO_2_ films appeared white (Fig. 6a), while after multiple cycles of uranium extraction from seawater, the film surface exhibited a pale yellow color (Fig. 6b), indirectly indicating significant uranium extraction. Electron dispersive spectroscopy (EDS) elemental mapping images revealed that the distribution of uranium (Fig. 5c) closely overlapped with that of titanium (Fig. 3d), suggesting that the adsorption of uranium by the substrate and binder was negligible, with the films primarily relying on catalysts for photocatalytic uranium extraction from seawater. Furthermore, XPS analysis of the {101} TiO_2_ after uranium extraction showed a noticeable peak around 380 eV binding energy in Fig. 6d, corresponding to U4f, compared to the wide-spectrum XPS profile of TiO_2_ without uranium extraction in Fig. 2b. This further confirmed the attachment of uranium at the catalytic interface. Meanwhile, peaks corresponding to other elements, such as Ti2p, showed no significant changes, indicating that the {101} TiO_2_ nanoparticles maintained relatively stable structural integrity during the process of uranium extraction from seawater. In addition, the peak shapes of the XRD patterns after uranium extraction from seawater (Fig. S3†) were similar to those before use (Fig. 2a), and they were all in the anatase crystal phase, which further verified the structural stability of the material.

**Fig. 7:**
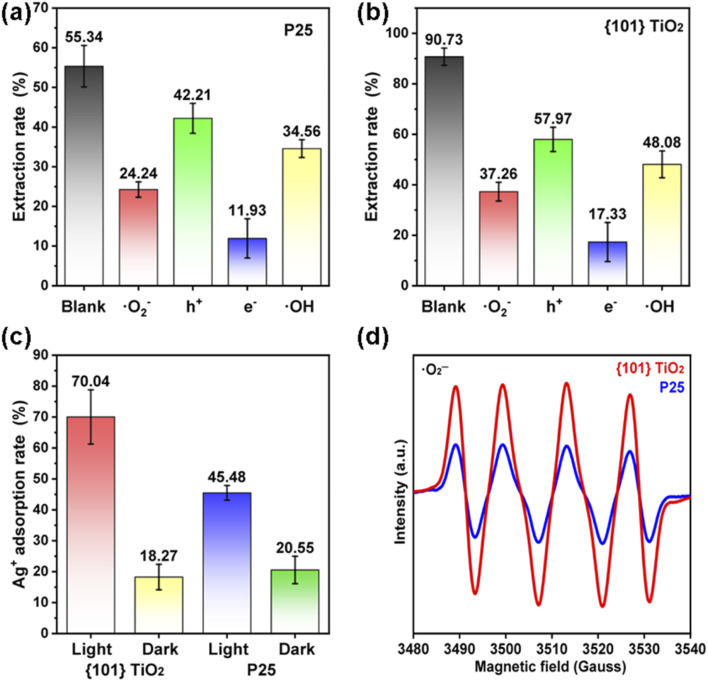
(a) Quenching experiments of active species on P25 films and (b) {101} TiO_2_ films. (c) Silver ion adsorption tests and (d) superoxide radical EPR tests for catalyst films.

The results indicate that the {101} TiO_2_ coating developed in this study is capable of efficiently and stably extracting uranium from seawater. To investigate the underlying mechanism behind its superior uranium extraction performance, the primary active species involved in the photocatalytic uranium extraction process were analyzed. By using 200 mg of ferric chloride, ammonium oxalate, *tert*-butanol, and *p*-benzoquinone to quench electrons, holes, hydroxyl radicals, and superoxide radicals, respectively, it was observed that the greater the reduction in uranium extraction efficiency after quenching, the more critical the corresponding active species were for uranium extraction. The results showed that the primary active species for both P25 coatings (Fig. 7a) and {101} TiO_2_ coatings (Fig. 7b) were superoxide radicals and photogenerated electrons. Subsequently, the generation of photogenerated electrons by P25 and {101} TiO_2_ coatings was analyzed through a silver ion adsorption test. Under dark conditions, the silver ion adsorption rates of both P25 and {101} TiO_2_ coatings were approximately 20% (Fig. 7c). Under light irradiation, the silver ion adsorption rates increased to 45.48% and 70.04% for P25 and {101} TiO_2_ coatings, respectively, representing increases of 24.93% and 51.77% compared to dark conditions. This increase in adsorption reflects the reductive effect of photogenerated electrons. Furthermore, electron paramagnetic resonance (EPR) analysis revealed that {101} TiO_2_ coatings generated a higher amount of superoxide radicals under light irradiation (Fig. 7d). In summary, photogenerated electrons and superoxide radicals are the key active species for uranium extraction from seawater. The superior photocatalytic uranium extraction performance of {101} TiO_2_ coatings can be attributed to their ability to generate a greater quantity of photogenerated electrons and superoxide radicals.

**Fig. 8:**
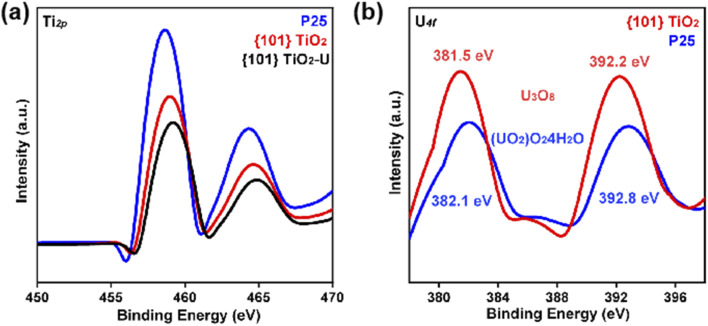
XPS spectra of titanium (a) and uranium (b) after uranium extraction by P25 and {101} TiO_2_.

Based on the above results, this study further investigated the underlying mechanism by which the {101} TiO_2_ coating generates a higher amount of reductive active species. The analysis of the titanium spectra obtained from X-ray photoelectron spectroscopy (XPS) revealed that the binding state between the titanium nucleus and its outer electrons reflects the tendency of the nucleus to lose electrons. A higher binding energy indicates a stronger tendency for electron loss. As shown in Fig. 8a, the binding energy of titanium in {101} TiO_2_ is higher than that in P25, suggesting that {101} TiO_2_ exhibits a stronger tendency to lose electrons compared to P25, making it more prone to interfacial reduction reactions. Furthermore, the increase in binding energy observed in {101} TiO_2_ after uranium extraction indicates the injection of electrons from {101} TiO_2_ into uranium species.

In addition, XPS analysis of the binding energies of the uranium elements shows that the uranium species formed on the {101} TiO_2_ surface have lower binding energies (Fig. 8b), indicating that they are in a deeper reduced state, a finding that is consistent with the results of the titanium spectroscopy analyses. Based on the specific binding energy results, it is known that the uranium species on the surfaces of {101} TiO_2_ and P25 are U_3_O_8_ and (UO_2_)O_2_4H_2_O, respectively, and combined with the quenching experiments of the reactive species in Fig. 7a and b, we can obtain the reaction eqn (1)–(3) for the extraction of uranium from seawater, which suggests that the {101} TiO_2_ has a stronger reducing property with sufficient electrons for the reduction of uranium from seawater, whereas the unmodified P25 surface has a low concentration of electrons for the indirect extraction of uranium *via* superoxide radicals. In summary, the ability of the {101} TiO_2_ coating to generate a higher amount of reductive active species can be attributed to the specific facet effects, which reduce the constraint of the titanium nucleus on its outer electrons. This reduction in constraint facilitates the excitation and release of electrons, thereby enhancing their participation in reduction reactions.

1 3Ca_2_UO_2_(CO_3_)_3_ + 6e^−^ + O_2_ → 6Ca^2+^ + U_3_O_8_ + 9CO_3_^2−^

2 O_2_ + e^−^ → ˙O_2_^−^

3 Ca_2_UO_2_(CO_3_)_3_ + 2̇O_2_^−^ + 4H_2_O → 2Ca^2+^ + (UO_2_)O_2_·4H_2_O(s) + 3CO_3_^2−^ + O_2_

## Conclusion

In summary, this study successfully developed a recyclable photocatalytic coating capable of efficiently extracting uranium from seawater. The results demonstrated that the exposure of reductive facets significantly enhanced the generation of reductive species, thereby effectively improving the efficiency of photocatalytic uranium reduction. Compared to unmodified coatings, the reductive coating exhibited notable advantages in uranium extraction rate, extraction capacity, and resistance to microbial contamination. Furthermore, the coating demonstrated adaptability to fluctuations in seawater conditions, including temperature, pH, and organic matter concentration, while maintaining stable performance over multiple cycles of reuse. This study provides valuable insights into the optimization of photocatalytic uranium extraction technology and its recyclability, further advancing its potential for practical applications.

## Conflicts of interest

There are no conflicts to declare.

## Supplementary Material

RA-015-D5RA02388B-s001

## Data Availability

All data supporting this study are openly available: experimental datasets (uranium extraction rates, adsorption capacities, and recycling performance) have been deposited in the Figure. Characterization data (XRD patterns, SEM/TEMimages, XPSspectra) are provided as ESI† associated with this article. Additional data related to anti-fouling performance and material synthesis protocols can be obtained from the corresponding author upon reasonable request.
