# Supplementary material for: Reductive faceted photocatalytic nanocoating for uranium extraction from seawater
Source: RSC Adv. 2025 Jul 23;15(32):26497–505. doi: 10.1039/d5ra02388b (PMC12285758; doi:10.1039/d5ra02388b)
Supplement: RA-015-D5RA02388B-s001 [file RA-015-D5RA02388B-s001.pdf]

Supplementary Information (SI) for RSC Advances.
This journal is © The Royal Society of Chemistry 2025

Reductive Faceted Photocatalytic Nanocoating for Uranium Extraction from Seawater

Chen Xie, Yizhi Zeng, Bohao Zhao, Ning Lv, Guiming Chen
(High-Tech Institute of Xi'an, Xi'an, Shaanxi, China, 710025）

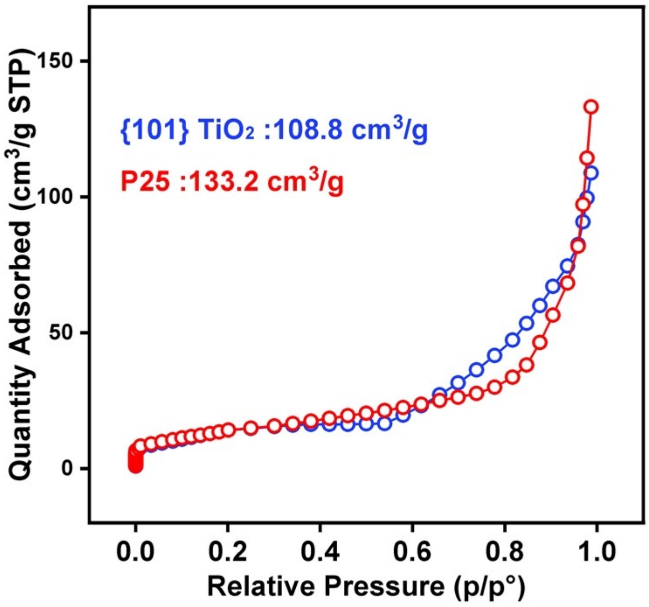

Figure S1. Specific surface area of two nanoparticles.

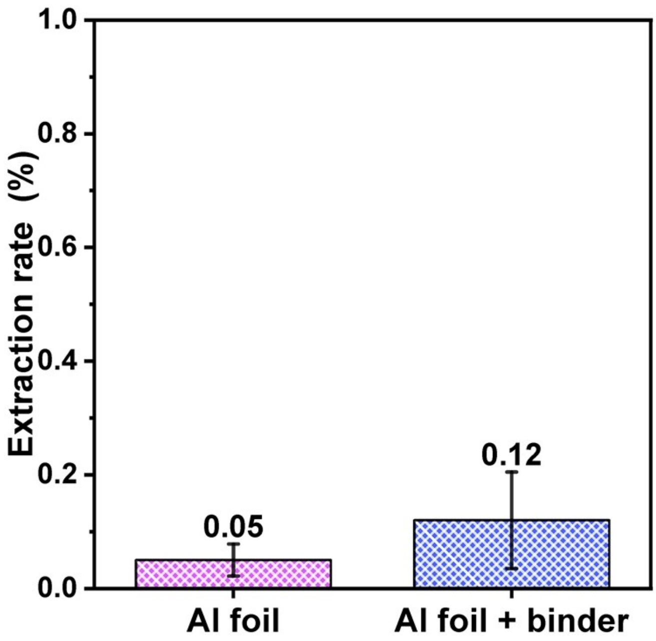

Figure S2 Effect of substrate on uranium adsorption.

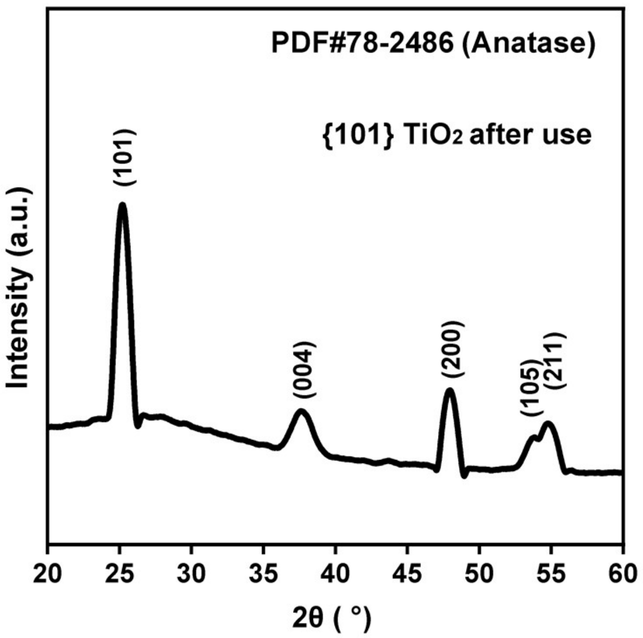

Figure S3 XRD pattern of {101} TiO_2 after uranium extraction from seawater.
